# Transcriptome Analysis of Moso Bamboo (*Phyllostachys edulis*) Reveals Candidate Genes Involved in Response to Dehydration and Cold Stresses

**DOI:** 10.3389/fpls.2022.960302

**Published:** 2022-07-19

**Authors:** Zhuo Huang, Peilei Zhu, Xiaojuan Zhong, Jiarui Qiu, Wenxin Xu, Li Song

**Affiliations:** College of Landscape Architecture, Sichuan Agricultural University, Chengdu, China

**Keywords:** moso bamboo (*Phyllostachys edulis*), abiotic stress response, transcriptome, dehydration, late embryogenesis abundant protein

## Abstract

Bamboo (Bambusoideae) belongs to the grass family (Poaceae) and has been utilized as one of the most important nontimber forest resources in the world. Moso bamboo (*Phyllostachys edulis*) is a large woody bamboo with high ecological and economic values. Global climate change brings potential challenges to the normal growth of moso bamboo, and hence its production. Despite the release of moso bamboo genome sequence, the knowledge on genome-wide responses to abiotic stress is still limited. In this study, we generated a transcriptome data set with respect to dehydration and cold responses of moso bamboo using RNA-seq technology. The differentially expressed genes (DEGs) under treatments of dehydration and cold stresses were identified. By combining comprehensive gene ontology (GO) analysis, time-series analysis, and co-expression analysis, candidate genes involved in dehydration and cold responses were identified, which encode abscisic acid (ABA)/water deficit stress (WDS)-induced protein, late embryogenesis abundant (LEA) protein, 9-cis-epoxycarotenoid dioxygenase (NCED), anti-oxidation enzymes, transcription factors, etc. Additionally, we used *PeLEA14*, a dehydration-induced gene encoding an “atypical” LEA protein, as an example to validate the function of the identified stress-related gene in tolerance to abiotic stresses, such as drought and salt. In this study, we provided a valuable genomic resource for future excavation of key genes involved in abiotic stress responses and genetic improvement of moso bamboo to meet the requirement for environmental resilience and sustainable production.

## Introduction

Bambusoideae, also called bamboo, belongs to the grass family (Poaceae) and is comprised of more than 1,400 species. Compared with other herbaceous species of Poaceae, the species of Bambusoideae are predominantly arborescent and perennial woody species. They can grow large woody culms up to 30 cm in diameter and 12 m in height (Barker et al., 2001) and are utilized as one of the most important nontimber forest resources in the world. According to the statistics, bamboo covers over 30 million hectares (ha) worldwide, and approximately 2.5 billion people depend economically on bamboo (Lobovikov, 2005), accounting for 68.8 billion US dollars in international trade in 2018 (International Bamboo and Rattan Organization).

Moso bamboo (*Phyllostachys edulis*) is a large woody bamboo with high ecological and economic values. It serves as a promising bio-resource for renewable forestry products and accounts for over two-thirds of the total bamboo growing area (4.43 million ha) in China (Peng et al., 2013). As sessile organisms, plants have evolved a wide spectrum of adaptations to cope with the inevitable challenges from environmental stress, such as drought, high salinity, and cold. Many aspects of these adaptation processes, including developmental, physiological, and biochemical changes, are regulated or achieved by stress-responsive gene expression (Huang et al., 2016a). Therefore, the identification of key genes involved in abiotic stress response is essential for the dissection of the complex mechanism underlying the stress tolerance, which will provide guidance for plant genetic improvement to meet continuous economic requirements and environmental resilience.

The draft genome sequence of moso bamboo was released previously (Peng et al., 2013), and an updated chromosomal level reference genome was also reported recently (Zhao et al., 2018). These genomic resources provide an opportunity for the excavation of stress-related genes at a genome-wide level. Huang et al. (2016a,b) analyzed genes encoding TIFY transcription factor and late embryogenesis abundant protein families in the moso bamboo genome and identified some stress-responsive genes. Jin et al. (2020) identified the expansin (EX) gene family in the moso bamboo genome and found that the expression of some *PeEXs* was induced by abscisic acid (ABA) and polyethylene glycol (PEG) treatments. Studies on stress-related functional genes have gradually increased. A moso bamboo WRKY gene *PeWRKY83* confers salinity tolerance in transgenic *Arabidopsis* plants (Wu et al., 2017); overexpression of *PeVQ28* in *Arabidopsis* increased resistance to salt stress and enhanced sensitivity to ABA (Cheng et al., 2020); a moso bamboo homeodomain-leucine zipper (HD-Zip) transcription factor Phehdz1 positively regulates the drought stress response of transgenic rice (Gao et al., 2021). *Arabidopsis* overexpressing *PheWRKY50-1* showed higher resistance to stress than the wild type (WT) (Huang et al., 2022). These studies made insights into moso bamboo's responses to abiotic stress. However, the genome-wide data for abiotic stress responses is still lacking. Recently, transcriptome profiling was conducted and revealed the crucial biological pathways involved in the cold response in moso bamboo (Liu et al., 2020).

To generate more genomic resources for stress-responsive gene mining, in this study, RNA-seq was employed to analyze transcriptomal responses of moso bamboo to dehydration and cold stresses. Differentially expressed genes (DEGs) at different time points of treatment were identified, and comprehensive stress-responsive gene exploration was also performed. A dehydration-responsive gene *PeLEA14*, encoding an “atypical” late embryogenesis abundant (LEA) protein, was selected as a tester to preliminarily investigate its function in drought and salt tolerance.

## Materials and Methods

### Plant Materials and Growth Condition

The *P. edulis* plants used in this study were approximately 2-year-old plants of *P. edulis*, which were manually planted and grown under the natural condition at Linyanshan Experimental Base (N31°00 33.2000, E103°360 51.9500) of Sichuan Agricultural University, Dujiangyan, Sichuan, China. The approximately 20 cM branch containing young unexpanded leaves of 4–5 centimeters long was cut from five plants in similar growth status. All these samples were collected in the morning (at approximately 10 o'clock and it was cloudy with a temperature of ~22°C). *Nicotiana tabacum* L. was used for *Agrobacterium*-mediated genetic transformation using the leaf disk method.

### Stress Treatments, Sampling, and RNA-seq

For dehydration treatment, the branches were placed on the dry filter paper and treated under room temperature (20°C and ~50% humidity). For cold treatment, the branches were put into a dark chamber set to 0°C. At different time points of treatments (2, 4, and 8 h for dehydration treatment, and 2 h and 4 h for cold treatment), ten unexpanded leaves were detached from the base and immediately frozen in liquid nitrogen and stored in the refrigerator at −80°C. The same amount of untreated leaves was also sampled and processed, which were used as control. One biological repeat for all samples was collected.

The total RNA was extracted according to the manual of the TRIZOL RNA Kit (TIANGEN, Beijing, China). The qualities and quantities of extracted nucleotide were measured using the NanoDrop 2000 Spectrophotometer (Thermo Fisher, USA) and the Agilent 2100 RNA 6000 Nano kit. The threshold of the quality of extracted RNA was RIN ≥ 7 with a concentration of ≥ 150 ng/μl and an amount of ≥ 5 μg. The cDNA library construction and pair-end sequencing on Illumina HiSeq^TM^ 4000 platform were performed by Onmath Co. (Chengdu, China), following the manufacturer's standard protocol.

### Quantification of Gene Expression Levels

The eXpress was used for transcripts quantification. An eXpress is a streaming tool for quantifying the abundances of a set of target sequences from sampled subsequences. A probabilistic model developed for RNA-seq is the underlying model of eXpress. In general, clean reads were aligned to reference transcripts of moso bamboo genome, and quantification was conducted based on alignments with the probabilistic model. As a result, estimated read count and transcript per million (TPM) $(TPM_{i}=\frac{q_{i\;/}l_{i}}{\sum_{{}_{j}}\left(q_{j/}l_{j}\right)}*10^{6}$, in which **q**_**i**_ indicates reads mapped to the transcript, **l**_**i**_indicates the transcript length, and **∑_j_(qj/lj)** denotes the sum of mapped reads to transcript normalized by transcript length), were obtained for every single transcript in every sample, even for the multi-isoform from the same gene.

### Differential Expression Test

A differential expression test was conducted using DESeq R packages according to the packages manual. Raw count data were prepared using the custom perl script based on results of eXpress software and were imported into the DESeq framework. Information on experiment design was also imported into the DESeq framework to form a Count Data Set. Filtering was performed to remove transcripts in the lowest 40% quantile of the overall sum of counts (irrespective of biological condition) to increase the differential expression transcript detection rate. The estimated SizeFactors function was used to estimate the effective library size in order to normalize the transcripts counts. The estimate Dispersions function was used to estimate dispersion. The nbinomTest function was used to see whether there is a differential expression between two conditions. FDRs were controlled using the Benjamini-Hochberg method (Benjamini and Hochberg, 1995).

### Gene Ontology Enrichment Analysis

Gene ontology (GO) enrichment analysis of the DEGs was implemented using the GOseq R packages based on Wallenius noncentral hypergeometric distribution (Young et al., 2010), which can adjust for gene length bias in DEGs.

### Plasmid Construction and Transformation in *N. tabacum*

Based on the coding sequence of *PeLEA14* (PH01001932G0350), a pair of primers, LEA-F: 5′-CCAAGCTTATGGCGCAGCTGATGGACAA-3′ (*Hind*III) and LEA-R: 5′-CGGGATCCTTAGAAGATGGTGGAGAGCGT-3′ (*Bam*HI), containing restriction enzyme sites (underlined), were designed to amplify the coding sequence of *PeLEA14* from cDNA using Phanta Max Super-Fidelity DNA Polymerase (Vazyme Biotech Co., Nanjing, China). The amplified fragment was double-digested and ligated onto the corresponding sites of the pGSA-1403 vector by T4 DNA ligase. The resulting construct 35S::pGSA1403-PeLEA14 was introduced into the *Agrobacterium tumefaciens* strain GV3101 and then transformed into *N. tabacum* using the leaf disk method.

The T_0_ seedlings were screened on 1/2 MS medium supplied with kanamycin (50 μg/ml). The seedlings resistant to kanamycin were transplanted into pots with soil, and the positive transgenic plants were further verified by PCR. The T3 homozygous positive lines were used for further investigation.

### Evaluation of Abiotic Stress Tolerance

For stress tolerance assays at the seedling stage, the WT and transgenic seedlings were placed in cultivation bottles with 1/2 MS solid medium containing 200 mM mannitol and 100 mM NaCl, respectively. The cultivation bottles were incubated with a cycle of 16 h/8 h of light (24°C)/dark (22°C).

For stress tolerance assays at the mature stage, 3-day acclimatization was performed for the WT and transgenic seedlings (with 4-5 leaves) grown on a 1/2 MS solid medium. Then, the plants were transplanted into pots (one plant per pot) containing an equal amount of sterilized soil and grown in an incubator with a cycle of 16 h/8 h of light (24°C)/dark (22°C). After 7 days, the plants with a similar growth status were selected for stress treatment. For natural drought treatment, the soil was fully watered, and then the watering was stopped for several days. For salt treatment, the soil was fully infiltrated with water, and then the plants were irrigated by applying enough 300 mM NaCl solution into the tray of cultivation pots and keeping the soil moist during the processing. The morphological changes of the plants were constantly observed and photographed.

### Measurements of Physiological Parameters Related to Stress Responses

Chlorophyll was extracted from leaf tissue in 95% ethanol as previously described (Palta, 1990). Proline was measured following the modified method of acidic ninhydrin reaction as reported previously (Bates et al., 1973). The enzyme liquid was extracted for the determination of superoxide dismutase (SOD), peroxidase (POD), and catalase (CAT) activities as well as malondialdehyde (MDA) content. Detailed descriptions of these assays were elaborated by Du and Bramlage (1992) and Zheng et al. (2007). Three replicates were executed for these experiments. Samples used for physiological index measurements were obtained through drought treatment (withhold watering) for 10 days and salt treatment (300 mM NaCl) for 7 days, respectively.

## Results and Discussion

### RNA Sequencing, Mapping, and Transcript Quantification

To understand transcriptomic responses of moso bamboo to abiotic stresses, RNA-seq analysis of bamboo leaves under dehydration and cold stresses was performed. We generated 33,498,161 to 58,049,004 clean reads for each sample by pair-end sequencing, corresponding to approximately 5.02 to 8.71 giga base pairs (Gb), with an average of 6.48 Gb (Supplementary Table S1). All clean reads were mapped to moso bamboo genome (Peng et al., 2013). Notably, ~66.6 to 71.9% of clean reads could be uniquely mapped and used for further analysis.

**Figure 1:**
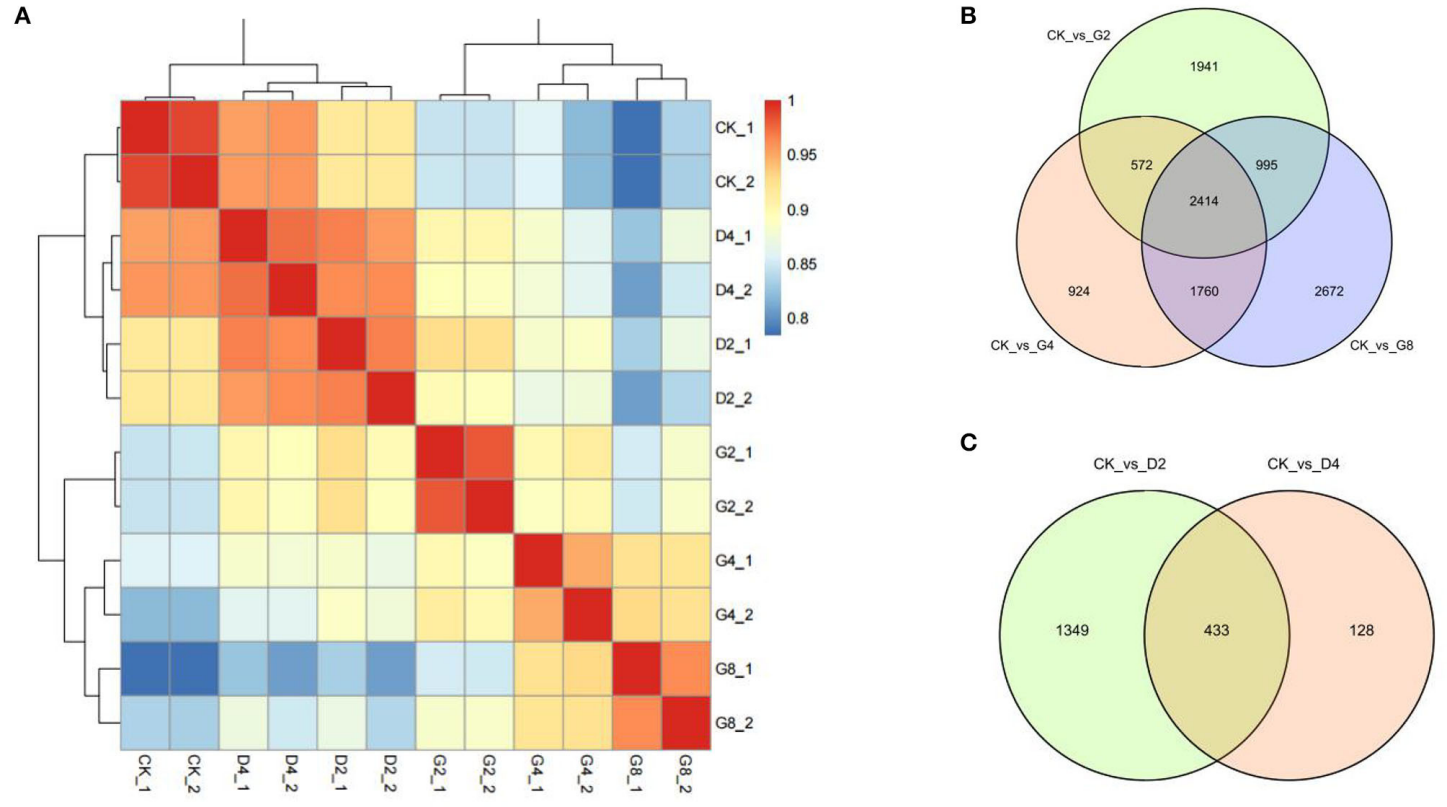
RNA-seq of moso bamboo leaves under dehydration and cold treatments. **(A)** Correlation and clustering of each sample. **(B,C)** Venn diagram of differentially expressed genes (DEGs) under two treatments. CK, control; D2 and D4 indicated 2 h and 4 h of cold treatment, and G2, G4, and G8 indicated 2 h, 4 h, and 8 h of dehydration treatment, respectively.

Subsequently, the transcripts per million (TPM) value was calculated to quantify the transcript abundance in each sample. To evaluate the repeatability of biological repeats, the correlation coefficient among samples was calculated based on the TPM values. The results showed that the correlation coefficient among the two biological repeats was more than 0.9, which was significantly higher than between other samples (Figure 1A). The cluster analysis also showed that the two biological repeats of each treatment were clustered together and separated from other samples (Figure 1A). These results suggested that the obtained RNA-seq data have good repeatability, providing a guarantee for subsequent data mining.

### Identification of DEGs Related to Dehydration and Cold Responses

We used false-discovery rate (FDR)-corrected *p*-value (adjusted *p*-value, padj), which was defined by Benjamini and Hochberg (1995), to identify DEGs between treatments. When set padj <0.01 as the threshold, 661 to 10,054 DEGs between CK and each dehydration and cold treatment were obtained (Figures 1B,C; Supplementary Figure S1). For three dehydration time points (2 h, 4 h, and 8 h), 10,054, 5,967, and 8,398 DEGs were identified, in which 5,388, 2,784, and 4,109 genes were upregulated, and 4,666, 3,183, and 4,289 genes were downregulated, respectively (Figure 1B, Supplementary Figures S2A,B). Notably, 1,364 and 1,027 genes were upregulated or downregulated at all time points of dehydration (Supplementary Figures S2A,B). For cold treatments, 2,322 and 661 DEGs were found at 2 h and 4 h of cold treatment, in which 1,418 and 565 DEGs were upregulated, and 904 and 96 DEGs were downregulated, respectively. A total of 433 DEGs showed differential expression at all two time points (Figure 1C, Supplementary Figures S2C,D). We noticed that the DEG number under cold treatment was much less than that obtained under dehydration. This phenomenon was also found in the previous study on transcriptomic responses to cold of moso bamboo (Liu et al., 2020). They only found <100 DEGs at early stages (0.5 and 1 h) of cold treatment (0.5 and 1 h).

### Functional Annotation of DEGs by GO Enrichment

**Figure 2:**
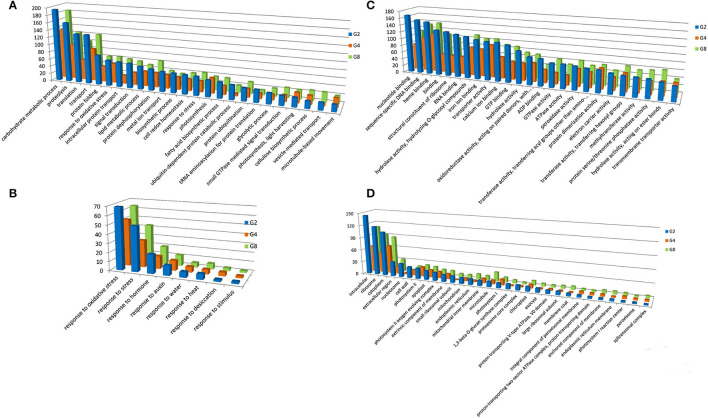
Gene ontology (GO) terms with predominant DEG number under dehydration treatment. GO terms of BP **(A)**, MF **(C)**, and CC **(D)** were shown. **(B)** Indicates GO terms of MF, which are closely related to stress responses. Y-axis indicated the DEG number assigned in a GO term. CK, control; G2, G4, and G8 indicated 2 h, 4 h, and 8 h of dehydration treatment, respectively.

We analyzed GO classification to categorize the functions of DEGs during dehydration and cold stresses (Supplementary Table S2). GO includes three main ontologies, namely, molecular function (MF), biological process (BP), and cellular component (CC). Under dehydration stress, the GO terms of BP in which the DEGs enriched are related to some basic BPs, such as carbohydrate metabolic process, proteolysis, translation, and transport, as well as those closely related to stress responses, such as response to oxidative stress, signal transduction, cell redox homeostasis, and response to stress (Figures 2A,B). For the MF, the predominant GO terms are nucleotide-binding, sequence-specific DNA binding, heme binding, binding, structural constituent of ribosome, RNA binding, hydrolase activity, hydrolyzing O-glycosyl compounds, iron ion binding, transporter activity, calcium ion binding, etc. (Figure 2C). For the CC, intracellular, ribosome, cytoplasm, extracellular region, nucleosome, cell wall, and photosystem II are among the most DEG-enriched GO terms (Figure 2D).

**Figure 3:**
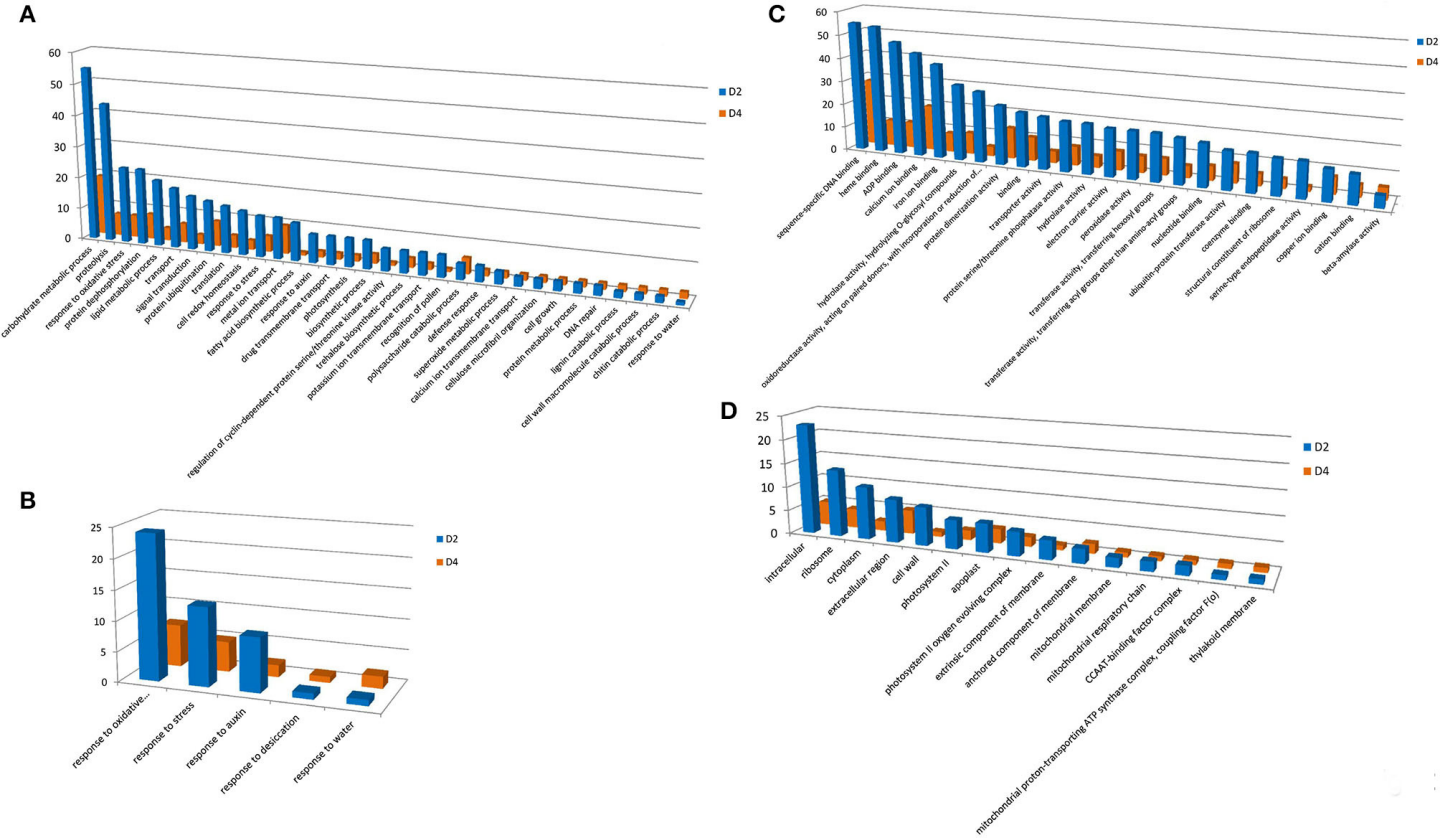
Gene ontology terms with predominant DEG number under cold treatment. GO terms of BP **(A)**, MF **(C)**, and CC **(D)** were shown. **(B)** Indicates GO terms of MF, which are closely related to stress responses. Y-axis indicated the DEG number assigned in a GO term. CK, control; D2 and D4 indicated 2 h and 4 h of cold treatment, respectively.

Under cold treatment, GO terms of BP containing a predominant number of DEGs are carbohydrate metabolic process, proteolysis, response to oxidative stress, protein dephosphorylation, lipid metabolic process, and transport (Figure 3A). There are five GO terms apparently related to environmental responses, i.e., response to oxidative stress, response to stress, response to auxin, response to desiccation, and response to water (Figure 3B). For the MF, DEGs were enriched in GO terms of sequence-specific DNA binding, heme binding, ADP binding, calcium ion binding, iron ion binding, hydrolase activity, oxidoreductase activity, protein dimerization activity, and binding (Figure 3C). For the CC, DEGs were enriched in GO terms of intracellular, ribosome, cytoplasm, extracellular region, cell wall, photosystem II, and apoplast (Figure 3D).

In previous cold-responsive transcriptomic studies, 89 and 79 DEGs were identified at 0.5 h and 1 h cold treatment, respectively, and 125 DEGs were annotated with GO terms. Among them, 21 DEGs were assigned GO terms of BP, including metal ion transport, response to UV-B, flavonoid biosynthetic process, response to light stimulus, response to salt stress, calcium ion transport, transcription, and response to salt stress. A total of 70 DEGs were assigned with MF GO terms. The GO terms with predominant DEG numbers included calcium ion binding (6 DEGs), DNA binding (11 DEGs), iron ion binding (5 DEGs), sequence-specific DNA binding (12 DEGs), protein serine/threonine kinase activity (3 DEGs), and protein serine/threonine phosphatase activity (DEGs). For the CC, the major GO terms were plastid (8 DEGs), integral component of membrane (5 DEGs), plasma membrane (4 DEGs), and cytoplasmic vesicle (3 DEGs). These results showed significantly different responses to cold at different time points. Interestingly, sequence-specific DNA binding, calcium ion binding, and iron ion binding are predominant GO terms in our and previous studies, indicating their general and important roles in cold responses of moso bamboo.

Differentially expressed genes enriched in these GO terms reflected transcriptomal responses of *P. edulis* to dehydration and cold stresses. For example, response to oxidative stress is one of the predominant GO terms under both stresses, indicating that oxidation is one of the most serious stresses under the two treatments (Supplementary Tables S2, S3). DEGs involved in this GO term mainly encode catalase, glutathione peroxidase, and a group of plant peroxidase (Supplementary Table S4). These enzymes were well known to scavenge excess reactive oxygen species (ROS) generated by stress (Zhang et al., 2019).

Response to stress is another common GO term. Under dehydration treatment, 24 DEGs enriched in this GO term were simultaneously found in three time points (Supplementary Table S5). Genes encoding ABA/water deficit stress (WDS)-induced protein, LEA protein (including dehydrin), and universal stress protein A (UspA) are among the most abundant (Supplementary Table S5). ABA/WDS-induced protein, also known as ASR, is a family of plant proteins induced by water deficit stress (Padmanabhan et al., 1997), or ABA stress and ripening (Canel et al., 1995) and has been extensively studied. The maize ZmASR1 acts as both a transcriptional regulator and a chaperone-like protein (Virlouvet et al., 2011). The rice *ASR5* was reported to be involved in response to drought stress by regulating ABA biosynthesis, promoting stomatal closure, and also acts as a chaperone-like protein possibly preventing drought stress-related proteins from inactivation (Li et al., 2017). LEA proteins are well known to play a protective role during exposure to different abiotic stresses. The Universal Stress Protein (USP) contains a Universal Stress Protein A domain comprising 140–160 highly conserved residues and is significantly overexpressed under multiple unfavorable environmental stresses (Kvint et al., 2002; Ndimba et al., 2005; Persson et al., 2007). Under cold treatment, four DEGs, PH01000696G0320, PH01001317G0160, PH01003240G0100, and PH01004855G0070, with GO term of response to stress were found in both time points (Supplementary Table S5). They encode ABA/WDS-induced protein, LEA protein 3, dehydrin, and calmodulin-binding protein-like (SARD1) (Supplementary Table S5) and also showed differential expression levels under dehydration treatment, indicating that these genes may play roles in both dehydration and cold stresses.

We also analyzed the DEGs enriched in sequence-specific DNA binding of GO category MF, as they usually include transcription factors. Crossing the three time points of dehydration treatment, 63 DEGs were assigned in this GO term. They all encode putative transcription factors, including 17 basic-leucine zipper (bZIP), 18 WRKY, six heat shock factor (HSF) type, 15 homeodomain, and seven GATA type (Supplementary Table S6). Under cold treatment, 25 DEGs enriched in sequence-specific DNA binding were detected in the two time points, including six bZIP, 18 WRKY, and one HSF (Supplementary Table S5). Eleven DEGs were simultaneously found in all time points of both treatments (Supplementary Tables S4, S5), indicating that they may play dual roles in responding to dehydration and cold stresses. Under unfavorable environmental conditions, plants have evolved diverse stress-response mechanisms, such as induction of defense gene expression. Therefore, activating the overall defense reaction ultimately contribute to stress response ad tolerance (Fraire-Velázquez et al., 2011). Transcription factors (TFs) are important regulators for the control of gene expression in all living organisms and play crucial roles in plant development, cell cycling, cell signaling, and stress response (Gonzalez, 2016). Extensive studies proved that TF families, such as AP2/ERF, MYB, NAC, and WRKY, are crucial regulators of various stress-responsive genes (Wang et al., 2016). Therefore, The TFs encoded DEGs identified in this study may help to obtain a better understanding of the mechanisms of abiotic stress response of moso bamboo and could be considered an ideal choice for genetic engineering in order to enhance stress tolerance.

### Time-Series Analysis of DEGs

**Figure 4:**
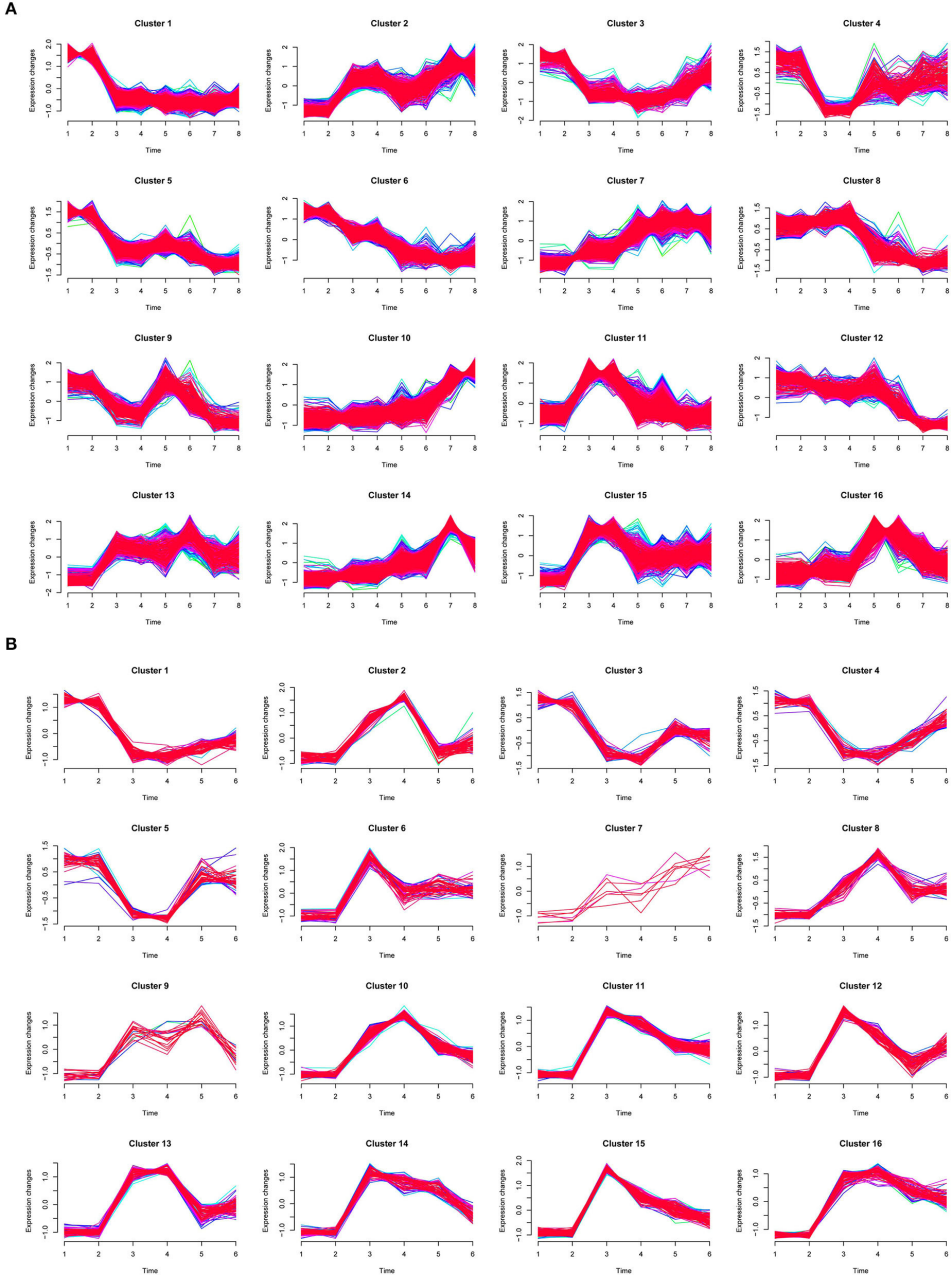
Time-series clustering of DEGs for dehydration **(A)** and cold **(B)** treatments. In **(A)**, 1 and 2, 3 and 4, 5 and 6, and 7 and 8 on *X*-axis indicated two biological repeats of CK, G2, G4, and G8, respectively. In **(B)**, 1 and 2, 3 and 4, and 5 and 6 on *X*-axis indicated two biological repeats of CK, D2, and D4, respectively.

To characterize dynamic expression patterns of DEGs following the time points of dehydration and cold treatments, an R package Mfuzz (Kumar and Futschik, 2007) was employed to perform time-series “soft clustering” based on TPM values (Figures 4A,B). DEGs with padj <0.01 between at least two time points were used as input for the clustering. The number of clusters was set to 16, and the fuzzifier coefficient was set to 1.55.

**Figure 5:**
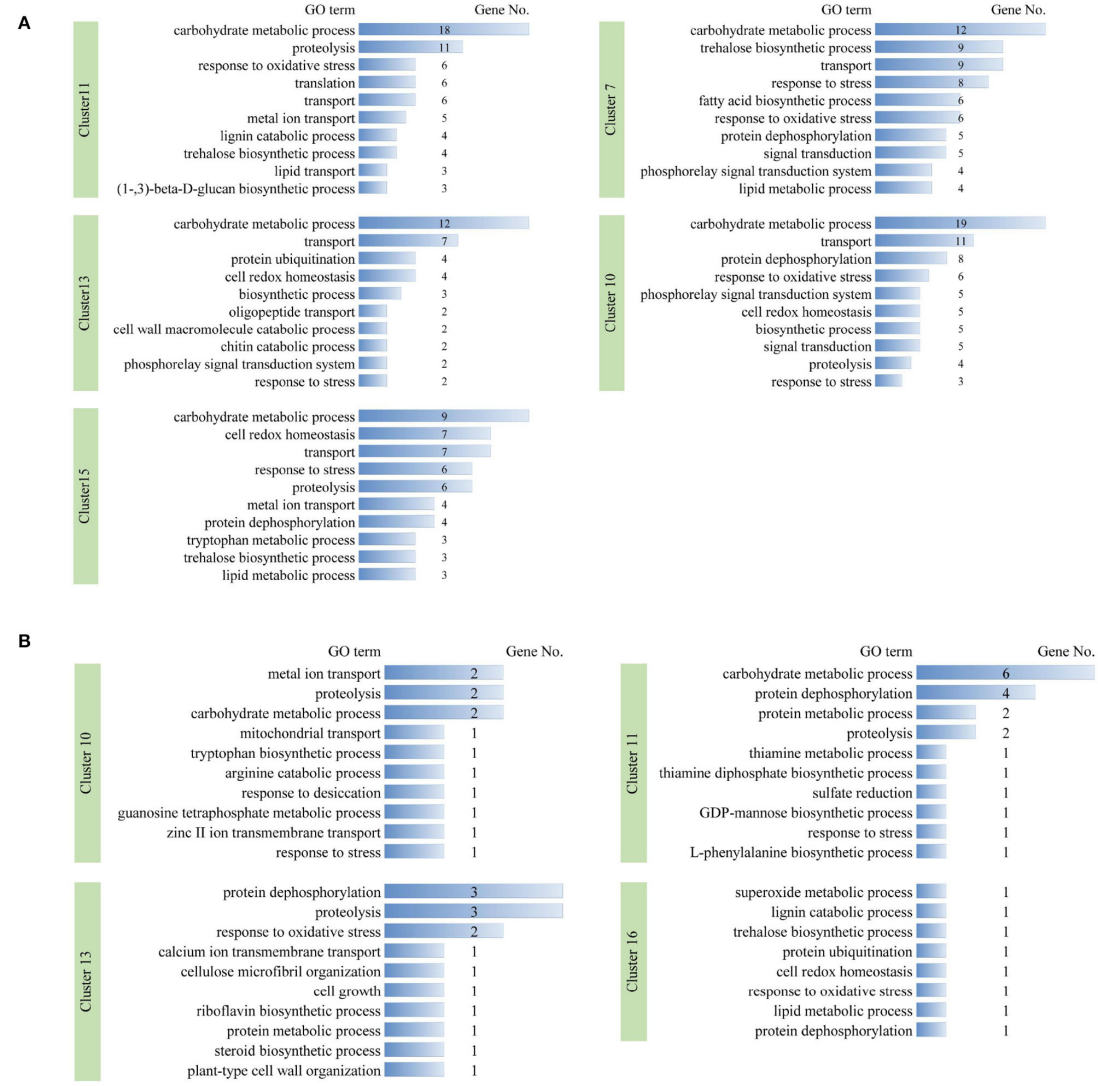
Gene ontology terms with predominant numbers of DEGs in stress-responsive timer-series clusters under dehydration **(A)** and cold **(B)** stresses, respectively. DEG number in each GO term is shown.

We focused on clusters in which the two biological repeats are at similar levels and showed stress-responsive patterns. For dehydration treatment, clusters 11, 13, and 15 showed a quick upregulation pattern (type I) at 2 h, whereas DEGs in clusters 7 and 10 were gradually upregulated (type II); clusters 1, 3, 4, and 5 exhibited early quick downregulation patterns (type III), and clusters 6 and 8 were gradually downregulated (type IV), respectively (Figure 4A). We further examined the functions of DEGs in upregulation clusters. GO terms with predominant gene numbers for type I and II clusters included carbohydrate metabolic process, proteolysis, response to oxidative stress, transport, protein ubiquitination, cell redox homeostasis, response to stress, trehalose biosynthetic process, and fatty acid biosynthetic process (Figure 5A). For cold treatment, clusters 10, 11, 13, and 16 showed type I upregulated patterns, and clusters 1 and 3 exhibited type III downregulated patterns (Figure 4B). GO terms for genes in these clusters included carbohydrate metabolic process, metal ion transport, protein dephosphorylation, proteolysis, and response to oxidative stress (Figure 5B). These data provided a resource to characterize gene sets showing similar patterns responsive to dehydration and cold stress.

### Identify Co-Expressed Hub Genes Based on Pearson'S Correlations

To identify potential key genes involved in dehydration and cold responses, we calculated Pearson's correlations for all gene pairs crossing all time points of two treatments. The gene filtering was performed with a cutoff value of 0.85, and the degree (number of associated genes fitting the cutoff) for each qualified gene was calculated. The top 50 genes were identified as potential hub genes, including those encoding 9-cis-epoxycarotenoid dioxygenase (NCED), LEA protein, probable indole-3-acetic acid-amido synthetase, protein phosphatase 2C, ubiquitin-activating enzyme, as well as bZIP, NAC, F-box, and SBP-box transcription factors (Supplementary Table S8). Some of these are well known to function in stress responses. For example, NCED is the key enzyme for the biosynthesis of ABAs (Hauser et al., 2011), which plays a critical role in abiotic stress response (Nambara and Marion-Poll, 2005). These results, therefore, provide candidate core genes, especially those with unknown functions, involved in the abiotic stress response of moso bamboo.

### Function Validation of Stress-Responsive Gene

**Figure 6:**
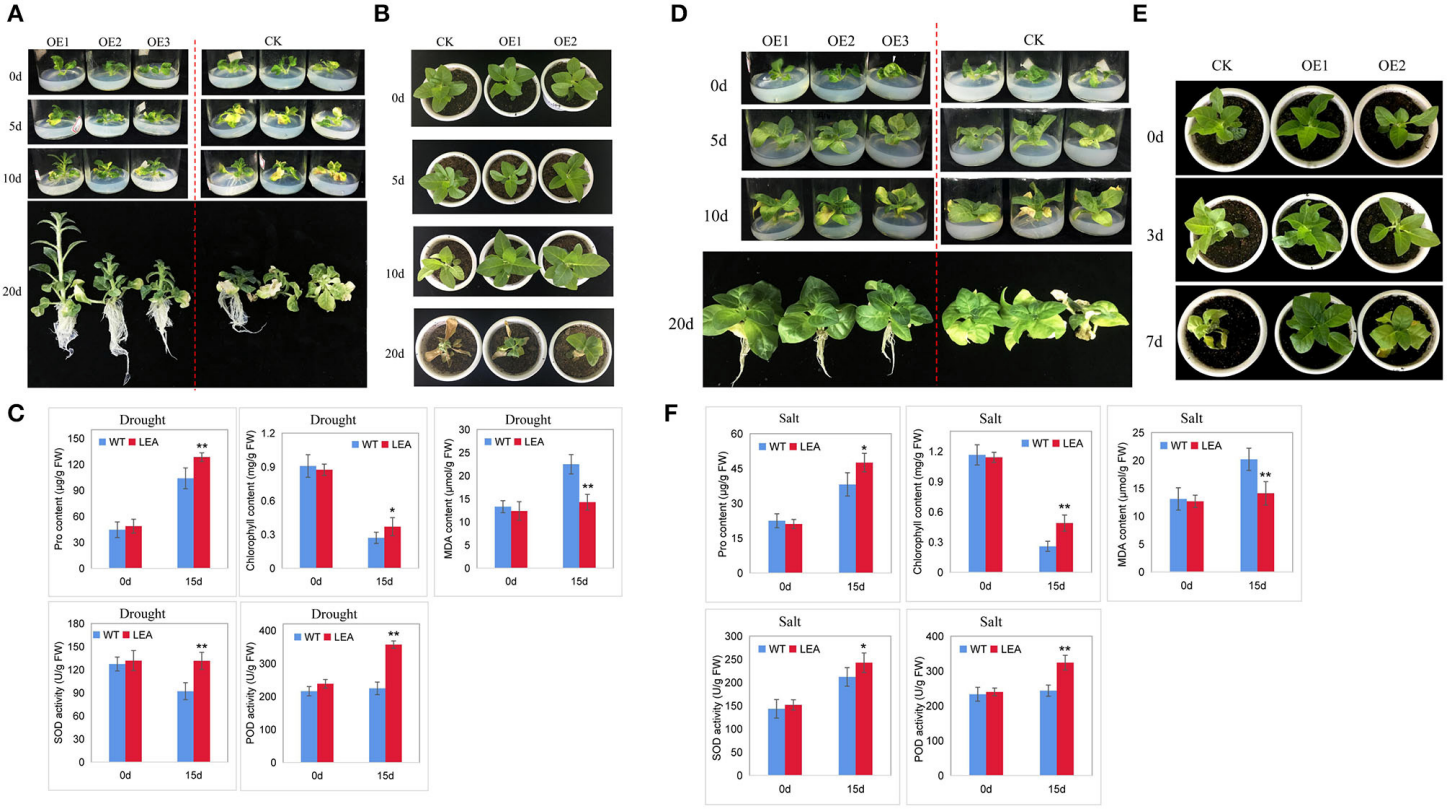
Overexpression of *PeLEA14* in tobacco. **(A–C)** indicate evaluation of tolerance of wild type (WT) and transgenic lines (OE) to drought stress at seedling (1/2 MS solid medium containing 200 mM mannitol) and adult (natural drought by withholding watering) stages and stress-related physiological parameters, respectively. **(D–F)** indicate evaluation of tolerance of WT and transgenic lines (OE) to salt stress at seedling (1/2 MS solid medium containing 100 mM NaCl) and adult (applying 300 mM NaCl solution into the tray) stages and stress-related physiological parameters, respectively.

To preliminarily test the effectiveness of the identified genes in abiotic stress tolerance, we selected a dehydration-responsive gene *PeLEA14* (PH01001932G0350) for functional validation. *PeLEA14* was slightly upregulated by cold but significantly induced by dehydration (Supplementary Figure S3). We overexpressed *PeLEA14* in tobacco. The positive transgenic lines were acquired from kanamycin resistance screening and PCR (Supplementary Figure S4). Artificially simulated drought treatment at the seedling stage was performed by growing plants on 1/2 MS solid medium containing 200 mM mannitol. The WT and transgenic lines were in similar status before treatment. After 5 days of treatment, the leaves of WT were slightly withered, and such symptoms became more serious by 10 days. At 20 days of treatment, almost all leaves of WT were withered, but the transgenic lines showed significantly better growth status and root system (Figure 6A). The natural drought treatment was also applied. Ten days after withholding water, the dehydration symptoms (chlorosis) could be observed on leaves of both WT and transgenic lines. However, the vegetative growth of the latter is better than that of the former. Twenty days after withholding water, the whole plant of WT was severely withered, whereas the growth status of transgenic lines was significantly better than WT (Figure 6B). We further evaluated physiological parameters related to stress responses. Under natural drought stress, the transgenic line exhibited significantly higher proline and chlorophyll content, lower MDA content, and higher SOD and POD activities (Figure 6C). We also evaluated tolerance to salt stress. Similar results to those under drought stresses were obtained and indicated that the tobacco lines overexpressing *PeLEA14* showed better growth performance under salt stress at both seedlings and adult stages, as well as higher antioxidant capacity (Figures 6D–F).

Late embryogenesis abundant proteins constitute a family of hydrophilic proteins that are presumed to play a protective role during exposure to different abiotic stresses. They were initially classified into six subgroups on the basis of specific domains (Dure et al., 1989), and different researchers have also tried to use different classification methods (Tunnacliffe and Wise, 2007; Battaglia et al., 2008; Bies-Ethève et al., 2008; Hundertmark and Hincha, 2008; Shih et al., 2008; Battaglia and Covarrubias, 2013). *PeLEA14* encodes 151 amino acids and is a homolog of *AtLEA14* (AT1G01470, *E*-value = 5e-63) of *Arabidopsis* and belongs to the LEA_2 group (also be classified as group 5 by a different nomenclature). The typical LEA proteins, such as those of groups 1, 2, 3, and 4, are genuine hydrophilic and share specific sequence motifs within each group. However, group 5 LEAs lack significant signature motifs or consensus sequences, contain a significantly higher proportion of hydrophobic residues than typical LEA proteins, and therefore were considered “atypical” LEA proteins (Baker et al., 1988; Galau et al., 1993; Battaglia et al., 2008).

Although this group has not been extensively investigated, some reports indicated that they will accumulate in response to diverse stresses in plants (Baker et al., 1988; Piatkowski et al., 1990; Maitra and Cushman, 1994; Zegzouti et al., 1997; Kimura et al., 2003). In this study, we found that *PeLEA14* was significantly induced by dehydration (Supplementary Figure S3). Additionally, a transgenic sweet potato that overexpressed *IbLEA14* showed increased tolerance to osmotic and salt stress by enhancing lignification (Park et al., 2011). Overexpression of *SiLEA14* of foxtail millet improved tolerance to salt and drought stresses (Wang et al., 2014). Overexpression of *OsLea14-A* in rice improved tolerance to dehydration, high salinity, CuSO_4_, and HgCl_2_ (Hu et al., 2019). Our study showed that the overexpression of *PeLEA14* enhanced the tolerance of tobacco to drought and salt stresses, possibly through, at least in part, increasing antioxidant capacity. These results indicated that the “atypical” LEA_2 group proteins may play important roles in plant stress protection. The data set provided in this study will lay a foundation for future discovery of stress-tolerant genes in moso bamboo.

## Conclusion

In this study, we generated an expression data set with respect to dehydration and cold responses of moso bamboo. With repeatable RNA-seq data and a strict screening cutoff, we identify a lot of DEGs. Combining comprehensive GO enrichment analysis, time-series analysis, and co-expression analysis, we identified DEGs closely related to dehydration and cold responses, stress-responsive DEGs with similar expression patterns, as well as potential core genes, which may play an important role in dehydration and/or cold stress. We used *PeLEA14* as an example to validate the function of the identified stress-related gene in tolerance to abiotic stresses, such as drought and salt. These data may be valuable for future excavation of key genes involved in abiotic stress responses and genetic improvement of moso bamboo.

## Data Availability Statement

All data present in this study can be found in the article or Supplementary Materials.

## Author Contributions

ZH: conceptualization, writing-original draft preparation, project administration, and funding acquisition. PZ and XZ: methodology. PZ and JQ: validation. WX and LS: data curation. All authors have read and agreed to the published version of the manuscript.

## Funding

This study was supported by the International Cooperation Project (2022YFH0066) funded by the Science and Technology Department of Sichuan Province, China, and the Shuangzhi Plan funded by the Sichuan Agricultural University. The funders had no role in the design of this study; in the collection, analyses, or interpretation of data; in the writing of the manuscript, or in the decision to publish the results.

## Conflict of Interest

The authors declare that the research was conducted in the absence of any commercial or financial relationships that could be construed as a potential conflict of interest.

## Publisher's Note

All claims expressed in this article are solely those of the authors and do not necessarily represent those of their affiliated organizations, or those of the publisher, the editors and the reviewers. Any product that may be evaluated in this article, or claim that may be made by its manufacturer, is not guaranteed or endorsed by the publisher.

## Supplementary Material

Supplementary Table S1 Statistics of RNA sequencing and mapping.

Supplementary Table S2 Gene ontology (GO) enrichment of differentially expressed genes (DEGs) under dehydration treatment.

Supplementary Table S3 Gene ontology enrichment of DEGs under cold treatment.

Supplementary Table S4 DEGs under dehydration treatment and enriched in response to oxidative stress.

Supplementary Table S5 DEGs under dehydration treatment and enriched in response to stress.

Supplementary Table S6 Consensus DEGs at three time points of dehydration and enriched in GO term Sequence-specific DNA binding.

Supplementary Table S7 Consensus DEGs at two time points of cold treatment and enriched in GO term sequence-specific DNA binding.

Supplementary Table S8 List of hub genes.

Supplementary Figure S1 Identification of differentially expressed genes (DEGs) at each of the time points.

Supplementary Figure S2 Venn diagram of upregulated and downregulated DEGs under dehydration and cold treatment.

Supplementary Figure S3 Expression patterns of *PeLEA14* under dehydration and cold treatments.

Supplementary Figure S4 Identification of positive transgenic lines by PCR. M, DNA marker. The amplified fragment is ~500 bp, which fits the length of *PeLEA14* (456 bp).
