# Supplementary figures and images for: Transcriptome Analysis of Moso Bamboo (Phyllostachys edulis) Reveals Candidate Genes Involved in Response to Dehydration and Cold Stresses
Source: Front Plant Sci. 2022 Jul 19;13:960302. doi: 10.3389/fpls.2022.960302 (PMC9343960; doi:10.3389/fpls.2022.960302)

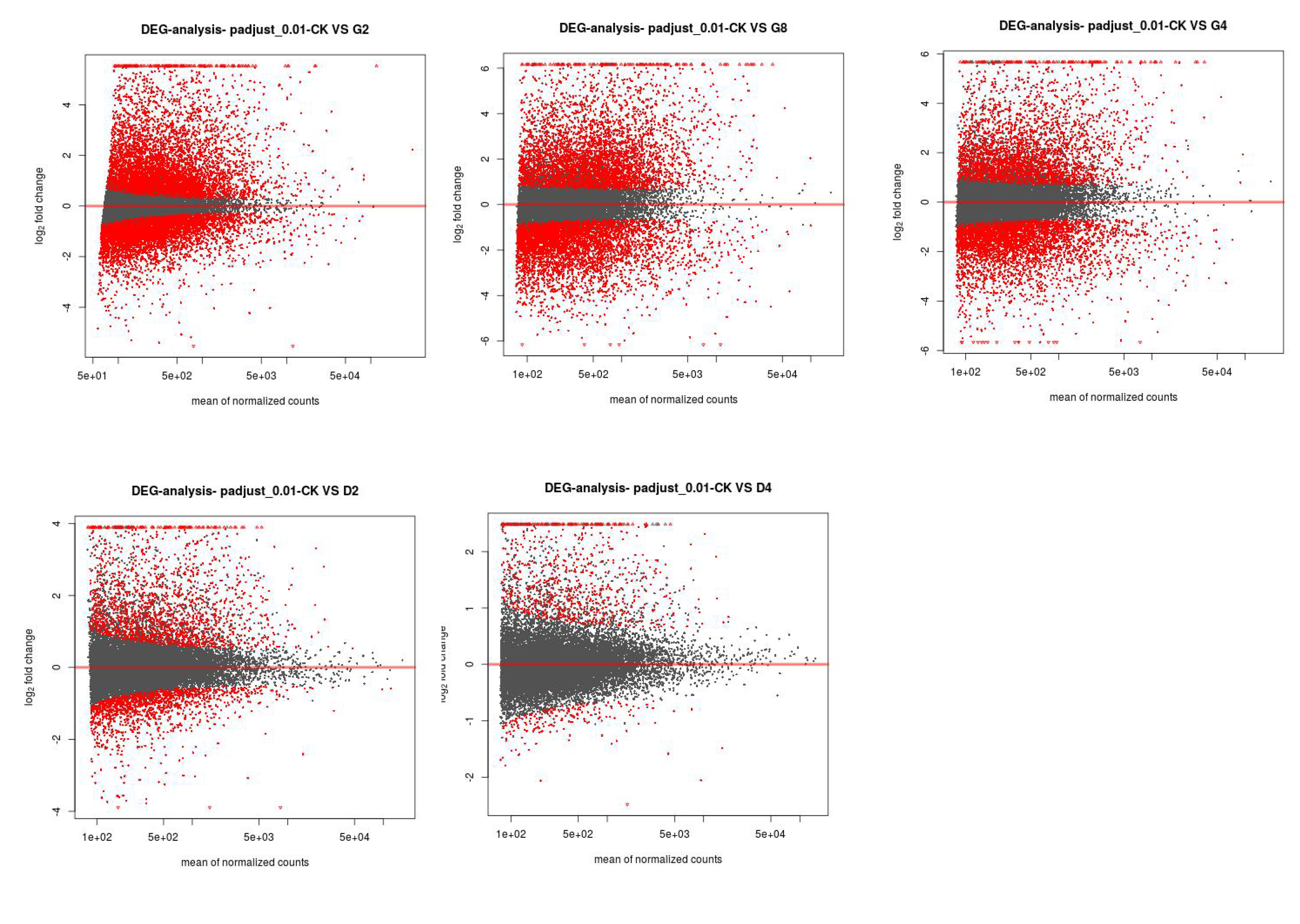

Supplement: Supplementary Figure S1 — Identification of differentially expressed genes (DEGs) at each of the time points. [file Data_Sheet_2.zip › Fig. S1.tif]

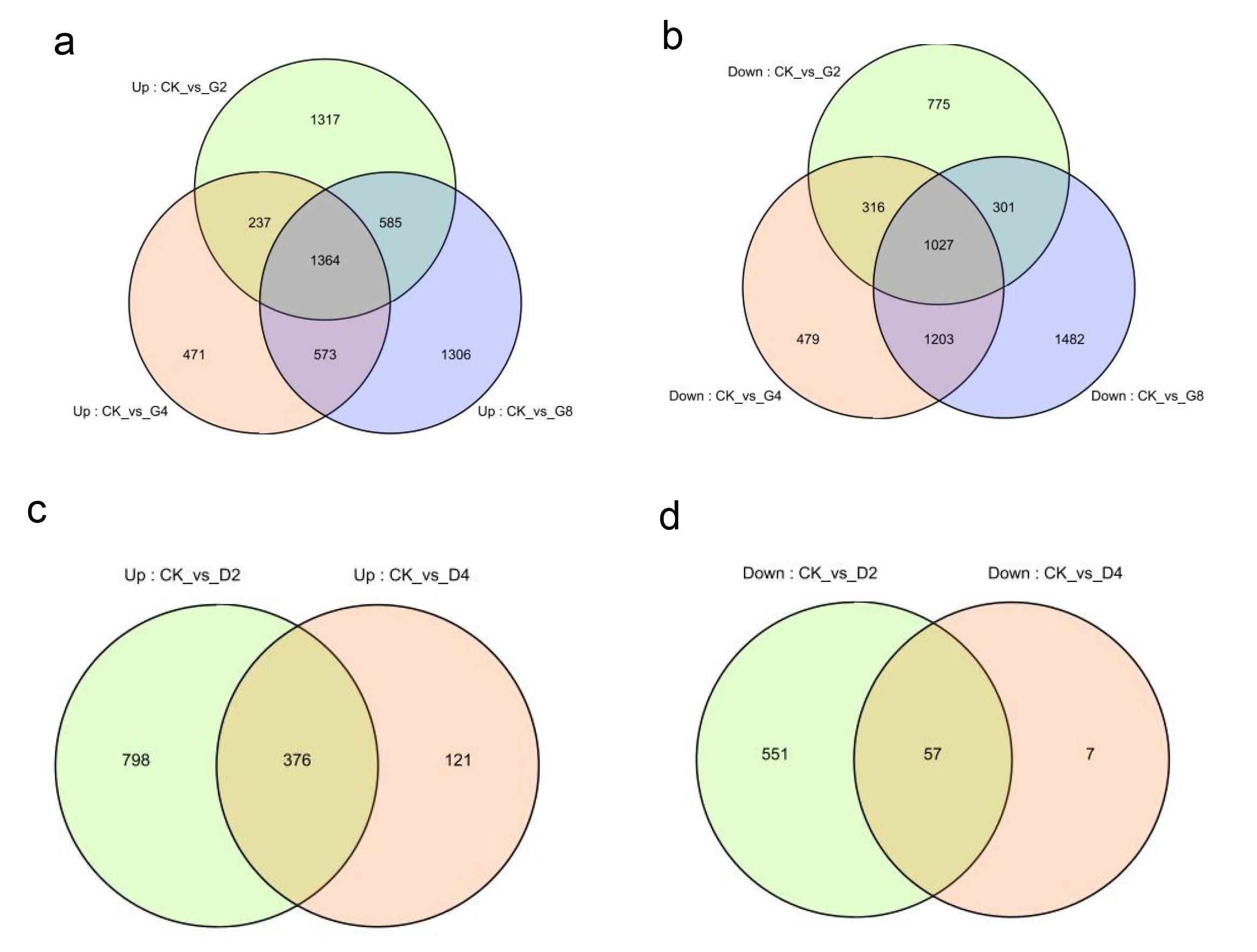

Supplement: Supplementary Figure S1 — Identification of differentially expressed genes (DEGs) at each of the time points. [file Data_Sheet_2.zip › Fig. S2.tif]

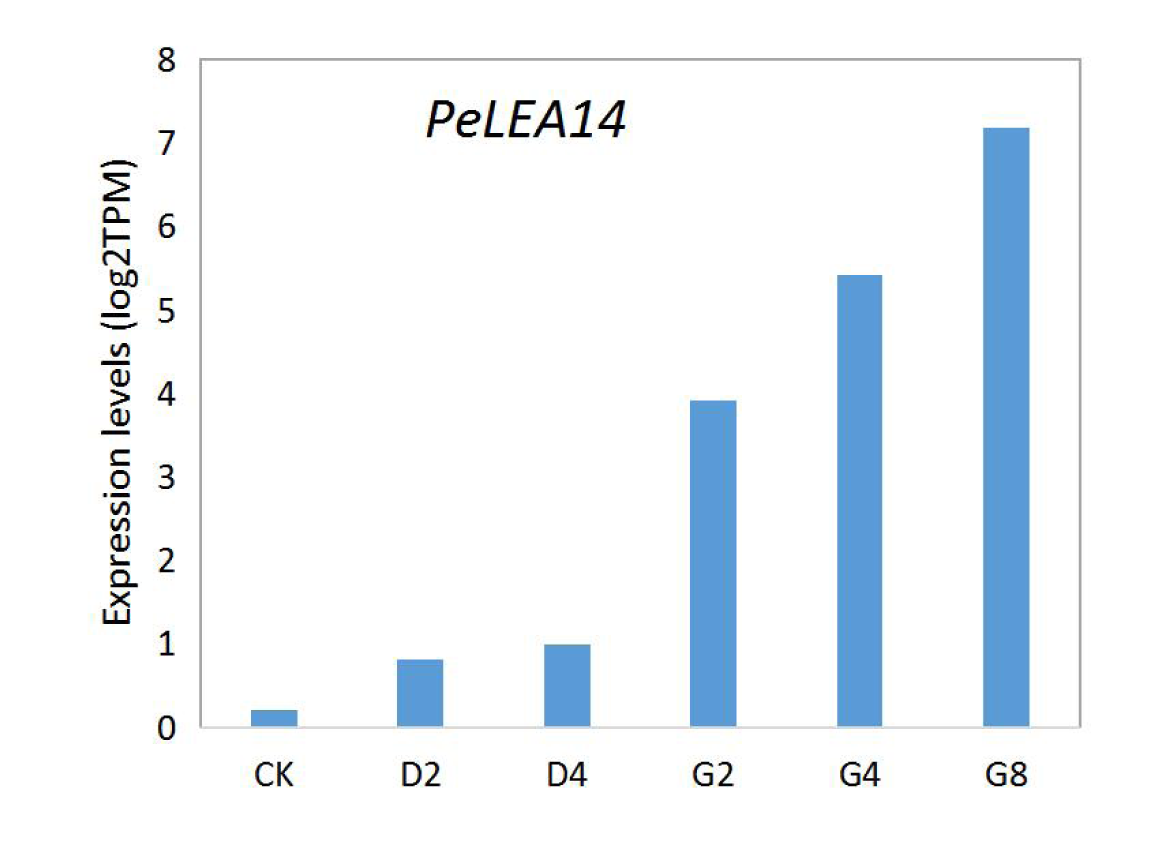

Supplement: Supplementary Figure S1 — Identification of differentially expressed genes (DEGs) at each of the time points. [file Data_Sheet_2.zip › Fig. S3.tif]

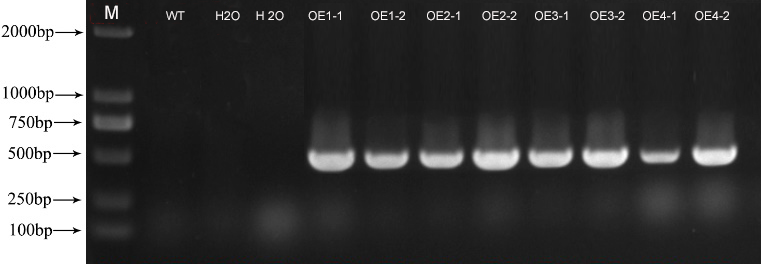

Supplement: Supplementary Figure S1 — Identification of differentially expressed genes (DEGs) at each of the time points. [file Data_Sheet_2.zip › Fig. S4.tif]
